# Heat Shock Transcription Factor 2 Is Significantly Involved in Neurodegenerative Diseases, Inflammatory Bowel Disease, Cancer, Male Infertility, and Fetal Alcohol Spectrum Disorder: The Novel Mechanisms of Several Severe Diseases

**DOI:** 10.3390/ijms232213763

**Published:** 2022-11-09

**Authors:** Yasuko Tokunaga, Ken-Ichiro Otsuyama, Shigeru Kakuta, Naoki Hayashida

**Affiliations:** 1 Department of Biochemistry and Molecular Biology, Graduate School of Medicine, Yamaguchi University, Yamaguchi 755-8505, Japan; 2 Institute of Gene Research, Yamaguchi University Science Research Center, Yamaguchi 755-8505, Japan; 3 Department of Clinical Laboratory Science, Faculty of Health Science, Graduate School of Medicine, Yamaguchi University, Yamaguchi 755-8505, Japan; 4 Laboratory of Biomedical Science, Graduate School of Agricultural and Life Sciences, The University of Tokyo, Tokyo 113-8657, Japan

**Keywords:** HSF2, transcription, complex, infertility, cancer, gene bookmarking, fetal alcohol spectrum disorder (FASD), neurodegenerative diseases (NDDs)

## Abstract

HSF (heat shock transcription factor or heat shock factor) was discovered as a transcription factor indispensable for heat shock response. Although four classical HSFs were discovered in mammals and two major HSFs, HSF1 and HSF2, were cloned in the same year of 1991, only HSF1 was intensively studied because HSF1 can give rise to heat shock response through the induction of various HSPs’ expression. On the other hand, HSF2 was not well studied for some time, which was probably due to an underestimate of HSF2 itself. Since the beginning of the 21st century, HSF2 research has progressed and many biologically significant functions of HSF2 have been revealed. For example, the roles of HSF2 in nervous system protection, inflammation, maintenance of mitosis and meiosis, and cancer cell survival and death have been gradually unveiled. However, we feel that the fact HSF2 has a relationship with various factors is not yet widely recognized; therefore, the biological significance of HSF2 has been underestimated. We strongly hope to widely communicate the significance of HSF2 to researchers and readers in broad research fields through this review. In addition, we also hope that many readers will have great interest in the molecular mechanism in which HSF2 acts as an active transcription factor and gene bookmarking mechanism of HSF2 during cell cycle progression, as is summarized in this review.

## 1. Introduction

All creatures, from yeasts to humans, maintain the homeostasis of their cells to survive. Sometimes they are exposed to drastic stresses including heat shock (heat stress), hypoxia, generation of misfolded protein, adenosine triphosphate (ATP) depletion, exposure to amino acid analogs, ethanol, oxidative stress, heavy metals, and pathological situations (fever, ageing, or neuronal injuries). Against these threats, cells have various protective mechanisms [1,2]. As one of the indispensable proteins for cellular protective mechanisms, HSF (heat shock transcription factor, or heat shock factor) has been discovered.

### 1.1. Classical HSFs, HSF1–4

HSF was identified as a transcription factor indispensable for heat shock response. Heat shock response is a cellular protective mechanism against heat stress discovered by Ritossa in 1962 [3,4,5,6]. While Ritossa was keeping larvae of *Drosophila melanogaster* at 25 degrees, he discovered that the specific region of the chromosome was puffing in the salivary gland of *Drosophila* under heat-stressed 37 degrees, indicating that some specific mRNA synthesis was upregulated [3,4]. Ten years later, Tissières and his colleagues showed that accelerated mRNA synthesis occurred in the heat shock protein (*HSP*) genes in the heat shock response [7,8].

It is notable that HSF was discovered as binding to a specific DNA sequence in the *HSP70* gene. In 1984, Wu found two regions at the 5′ end of *HSP82* gene in *Drosophila* (this gene is homologue of *hsp70* gene in mammals) which are resistant to exonuclease digestion. This result means that some proteins bind to these regions. With this result and other data, he reported that at least two protein factors bind to these regions and activate *hsp82* gene [9,10]. He named these protein factors as heat shock activators. Subsequently, he prepared cell extracts from heat-shocked cells and detected heat shock activator activity in these extracts by reconstituting specific binding of heat shock activator protein to the *HSP82* gene (homologue of *HSP70* gene in mammals) chromatin in vitro. This binding was inhibited by free DNA from the 5′ region of the *HSP82* gene.

This result indicated that the mechanism of HSP binding to the heat shock consensus sequence in the *HSP82* gene promoter is common among heat shock genes [11]. The heat shock consensus sequence in the *HSP82* gene promoter was named as heat shock elements (HSEs), and homologues of heat shock activator protein were discovered in other creatures and cells, and named as heat shock transcription factor (HSF).

Several groups were discovered in HSF protein including *Saccharomyces cerevisiae*, *Drosophila melanogaster*, and HeLa cell in the same year (1987) [12,13,14,15]. Moreover in 1991, cloning of both *HSF1* and *HSF2* were discovered in human and mouse studies. Formerly discovered *HSF* was named as *HSF1* and the other *HSF* was named as *HSF2* [6,16,17,18].

Human *HSF4* was discovered and cloned from HeLa cDNA library in 1997 [19], but more than 10 years were required to discover *HSF3*. Mouse *HSF3* was successfully cloned but human *HSF3* was found to be a pseudogene in 2010 [20].

### 1.2. The Importance of Non-Classical HSFs, HSF5, and HSFY, in Spermatogenesis and Fertility

Four classical (canonical) *HSF*s have been discovered with the turn described above. In addition, these classical *HSF*s were revealed to be involved in development and gametogenesis [21,22,23,24,25,26,27,28,29,30]; with this in mind, some researchers tried to discover new *HSFs*. In these cases, non-mammalian vertebrates were often used, for example, *HSF5* was discovered and cloned in zebrafish by Orban and his colleagues for the first time. They studied developmental processes using zebrafish and performed analysis on the gene sets expressed in the adult zebrafish testis and ovary to identify the genes with a potential role in fish gonad development and function [31]. Through this analysis, they identified *HSF5* with enhanced expression in adult zebrafish testis [32]. Moreover, they used the Crispr-Cas9 technique and generated *HSF5*-deficient zebrafish, and discovered that *HSF5*-deficient male zebrafish are infertile with a drastically reduced sperm count, increased sperm head size, and abnormal tail architecture. However, the females remained fertile [32].

On the other hand, Azarnia and his colleagues reported that the *HSF5* gene is expressed in early embryonic development and in adult testis spermatogonia and spermatocytes in mice [33]. If the *HSF5* surely exists in mouse testis, their discovery is very critical because it indicates that we may consider the *HSF5* gene for normal testis development and spermatogenesis in mammals including humans.

Male infertility plays a role in about half of infertility cases, and almost 30% have been thought to be recently caused by a male factor alone [34]; thus, the gene and its products can be the essential target for the development of medicine and therapy. As one problem, Azarnia and his colleagues reported HSF5 by only showing the immunohistochemistry analysis data using their original monoclonal antibody. We consider whether *HSF5* existence will be confirmed by Western blot, RT-PCR, and immunohistochemistry, or by other antibodies. Whether *HSF5* exists in mammalian testis is involved in normal testis development and spermatogenesis as well as zebrafish is very important; therefore, more detailed analysis is required.

As well as *HSF5*, other non-classical *HSF* genes called *HSFY* were demonstrated to exist on the human Y chromosome as multicopies by Nakahari and his colleagues in 2004 [35]. The human *HSFY* gene located on Yq has a long open reading frame containing an HSF-type DNA-binding domain (DBD) [6,35]. As a considerable problem, the amino-acid homology of DBD between HSFY and classical HSFs is very low; thus, it is not clear whether HSFY binds to the same DNA promoter sequence. For example, the DBD homology between HSFY and HSF1 is only 31% [35]. In addition, Foresta and his colleagues reported that this gene generates three different transcripts by alternative splicing and transcript 1 can be detected in brain, pancreas, testis, and sperm tissue in the same year 2004. However, this transcript cannot be detected in Sertoli cells. Transcripts 2 and 3 were detected only in testes [36].

*HSFY* was discovered in the same year by two different groups. It should be largely known that the deletion of *HSFY* was discovered from the men with azoospermia, oligospermia, and inspermatogenesis [35,36]. In 2012, Paduch and his colleagues found that the deletion or underexpression of *HSFY* is associated with maturation arrest in American men with nonobstructive azoospermia [37].

Now, that the importance of normal spermatogenesis commands considerable attention, which is much more than before. *HSF5* and *HSFY* studies will be required to proceed with a higher speed.

**Significance of Section 1:**

Heat shock response is a cellular protective mechanism against heat stress discovered by Ritossa in 1962.

*HSP*s were found as the genes of which mRNAs synthesis was increased in heat shock response.

Heat shock response is a pivotal role of HSF, but it can be induced by only HSF1, not by other HSFs.

Only HSF1 is activated by heat stress. HSF2–4 are not activated.

*HSF1* and *HSF2* were cloned in both human and mouse in the same year (1991).

Discovery and cloning of mouse *HSF3* followed in 2010, but the human *HSF3* is a pseudogene.

Non-classical *HSF*s, *HSF5,* and *HSFY* are probably required to be more intensively studied from the perspective of spermatogenesis and fertility.

## 2. HSF2 Research History in Early Period

### 2.1. HSF2 Is Activated by Various Stimuli but Not by Typical Heat Shock

Most of the early discoveries of HSF2 functions and characteristics come from the contribution of Morimoto and his colleagues. They examined several compounds and tried to find HSF-activating chemicals. Finally, they succeeded in discovering that hemin, a normal cellular metabolite iron-containing protoporphyrin, can activate HSF2.

Before publishing the discovery of hemin as an HSF2 activator, they reported that sodium salicylate, an anti-inflammatory agent, activated the DNA binding of HSF but did not induce heat shock gene transcription even though HSF was bound to the HSE of the *HSP70* promoter in HeLa cells [38]. In this paper, they did not show which HSF was activated by sodium salicylate. However, this report that the *HSP70* gene was not activated in spite of HSF being bound to the *HSP70* promoter should be considered as an important discovery.

In the same year, 1992, they subsequently reported that the DNA-binding activity of HSF2 is not activated by heat shock but is activated by hemin [39]. Hemin was already shown to be important for erythroid differentiation, in regulating translation initiation, catalyzing peroxidation reactions, and exerting a mitogenic effect on T cells [40,41,42,43]. The reason why Morimoto’s group used hemin is that they already found the increase in HSP70 synthesis upon treatment of K562 cells with hemin, based on the discovery of Singh and Yu [44].

While Morimoto and his colleagues published many HSF2 papers in the 1990s, they frequently used the hemin and human erythroid cell line, K562, because it was reported that heat shock-like protein (this protein is related to HSP70) was found to be accumulated during K562 differentiation [44]. The discovery of the activation mechanism of HSF2 was a big progress in HSF2 research.

Morimoto groups also discovered that other stimuli could activate HSF2. In 1998, they reported that the stress occurred by the inhibition of the ubiquitin–proteasome pathway activates HSF2 [45]. In the experiments performed in this study, they used various proteasome inhibitors including hemin, MG132, and lactacystin. In addition, they also used several cell lines, mouse ts85 breast cancer cells, human HeLa S3 cervical carcinoma cells, human HspG2 hepatocarcinoma cells, mouse embryonic fibroblast (MEF) cells, and human K562 erythroleukemia cells. It is likely that they did not show all the experimental data using these three proteasome inhibitors, at least, hemin and MG132 activate only HSF2 in K562 cells, and MG132 activates both HSF2 and HSF1 in MEF [45].

The mouse ts85 breast cancer cell has been hardly used in recent research, but this cell has a unique character caused by the mutation in ubiquitin-activating enzyme E1 [46,47]. Mouse ts85 cells were maintained at 30 degrees, when they were kept in heat-stressed 39.5 degrees, only HSF2 was activated and HSF1 was not [45]. HSF2 activation by heat stress does not usually occur, but it very rarely occurs in the distinctive charactered cells such as ts85 cells.

HSF2 can be activated by other stimuli. Tateishi and Iwaki’s group were investigating αB-crystallin (CRYAB), a well-known small HSP, for a long time, and found that the CRYAB protein is mainly expressed by astrocytic tumors among neuroectodermal neoplasms, frequently observed among astrocytic tumors, schwannomas, hemangioblastomas, and chordomas, and discovered that these pathological brain lesions cause an abnormal elevation in the potassium (K^+^) concentration of the extracellular space [48,49,50]. Moreover, they discovered that K^+^ induced CRYAB expression and that CRYAB expression in rat C6 glioma cells protect these cells against the insult of elevated extracellular K^+^ on the glioma cells [49]. However, they could not reveal the molecular mechanism for the induction of CRYAB in diseased brains and neuronal cells.

To reveal this mechanism, they used human U-251MG glioma cells and potassium chloride (KCl) to reproduce the abnormal elevation of K^+^ concentration. They succeeded in inducing CRYAB expression in U-251MG cells by KCl treatment [51].

Subsequently, they performed reporter assay using human *CRYAB* promoter containing a heat shock element (HSE) sequence, and revealed that this HSE sequence is indispensable for *CRYAB* induction. This was the strong evidence in which HSF is responsible for this induction. Finally, they identified that HSF2 is the essential factor for this molecular mechanism and that CRYAB is induced by a high concentration of K^+^ [51].

As they and other groups described, CRYAB induction is observed in the brain under various stress conditions or pathological situations [48,52,53,54,55]. CRYAB is also induced by heat stress through HSF1 activation; however, a high temperature state such as heat stress will nearly occur in the body.

In this point of view, the HSF2-CRYAB pathway and HSF2 activation in the cells, tissues, and organs are worth investigating for the development of therapeutics against various disorders.

### 2.2. HSF2 Is Involved in HSP70 and Other HSPs Induction

Described above, HSF2 cannot be activated by heat stress, but this fact does not mean that HSF2 cannot induce *HSP70* expression, either. Morimoto and his colleagues discovered that *HSP70* is also induced by HSF2 under hemin treatment [56]. In the experiments for this discovery, hemin treatment induced the nuclear translocation of HSF2, not HSF1, and HSP70 and HSP90 were also induced. Induction of HSP proteins including most major heat shock protein HSP70s has been recognized as a representative phenomenon of heat shock response; however, this report showed that the induction of HSPs was also caused by other stimuli. This discovery should be known as an important mechanism of HSP induction.

Wang and his colleagues reported that the overexpression of HSF2 conjugated with Enhanced Green Fluorescent Protein (EGFP) by transfection increases HSP27, HSP47, HSP70, and HSP90 proteins in both human normal lung epithelia BEAS-2B and human lung cancer A549 cells [57] (Table 1). This experimental system was artificial because they established the expression vector of human HSF2 and overexpressed it by transfection; thus, protein expression level of HSF2 was much higher than endogenous level. They also used cancer cells, but importantly, they did not give any stimuli to the transfected cells. Although there is one problem that they did not show the HSF1 protein level when HSF2 was overexpressed, the data that overexpressed HSF2 increased several HSPs’ protein expression from Wang’s group are valuable to show that HSF2 can induce several HSPs including HSP70.

### 2.3. HSF2 Is Expressed in Embryos and during Their Development

When embryos are kept and developed within the mother’s body, unless abnormal conditions prevail, it seems that activation of the mechanism against various stress including heat shock response is not necessary. In fact, heat shock response does not occur in all the cells produced during the life of a multicellular organism, e.g., early mouse embryos [58].

However, importantly, it was revealed that HSF2 is expressed in testis and during preimplantation development in mice [24]. HSF2 protein is expressed at least until 15.5 days of embryogenesis, and it is restricted to the central nervous system during the second half of gestation. HSF2 is expressed in the ventricular layer of the neural tube which contains mitotically active cells but not in postmitotic neurons. In addition, the HSF2 protein expression pattern did not coincide with four major HSPs, HSP90α, HSP90β, HSC70, and HSP25. Surprisingly, even *HSF2* mRNA expression was not identical to its protein expression [24].

Leppä and his colleagues examined HSF2 expression during mouse heart formation. During heart development, HSF1 showed abundant expression. However, in the HSF2 case, three different isoforms, alternatively spliced *HSF2α* and *HSF2β*, and an additional higher molecular weight isoform were strongly upregulated in the developing mouse heart at E11.5–12.5 [26]. *HSP* gene expression did not correlate with HSF2 in temporal or spatial.

The data from these two groups suggest that HSF2 expression is not involved in the regulation of inducible heat shock gene expression during development. These findings in the early period are important because they show the individuality of HSF2 of which roles must be different from HSF1.

**Significance of Section 2:**

HSF2 is activated by iron-containing protoporphyrin metabolite hemin, proteasome inhibitor MG132, and elevation of potassium concentration, but not by heat shock.

Hemin treatment can induce *HSP70* expression and high concentrations of potassium ion (K^+^) can induce small HSP *αB-crystallin* (*CRYAB*) through HSF2 activation.

Overexpression of HSF2 can increase some major *HSP*s expression including *HSP70*.

HSF2 is not involved in the inducible heat shock gene expression during development.

## 3. HSF2 Is Required for the Suppression of Polyglutamine Diseases Onset and Progression

In 2001, Zimarino and his colleagues overexpressed constitutive active HSF1 (caHSF1) which is deleted in its 202aa–316aa region (this region inhibits HSF1 activation) for the first time [59]. Our group successfully showed that HSF1 is required for the suppression of Huntington’s disease (HD), one of polyglutamine (polyQ) diseases, by crossing constitutive active HSF1 (caHSF1) or *HSF1*-deficient mice to R6/2 HD model mice [60,61].

**Figure 1:**
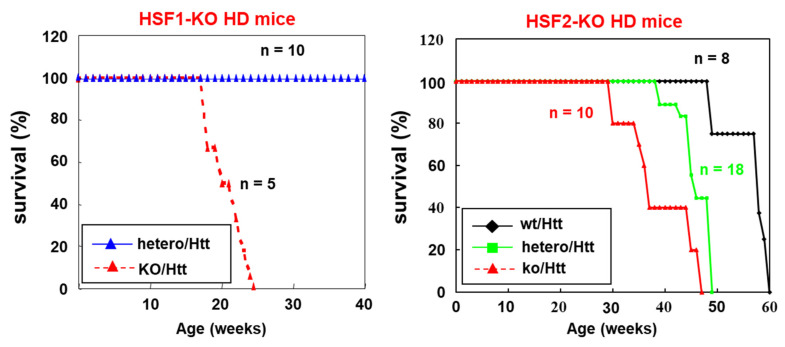
Deficiency of HSF1 and HSF2 causes the shortening of life span in Huntington’s disease (HD) mice. *HSF1*-KO HD mice died within 24 weeks but *HSF1*-hetero HD mice survive more than 40 weeks (**left**). *HSF2*-KO HD and *HSF2*-hetero HD mice died within 46 and 48 weeks (**right**), respectively. But *HSF2*-WT HD mice survived until 60 weeks.

Subsequently, we showed the importance of HSF2 in the HD suppression by the establishment of *HSF2*-KO HD mice as well as HSF1 experiments. HSF2-KO HD mice showed a prominent shortening of the life span [62]. Surprisingly, *HSF2*-hetero HD mice also showed life span shortening compared to *HSF2*-WT HD mice. These data showed a clear contrast to the data that *HSF1*-hetero HD mice did not show the shortening of life span and survived for the same period as non-HD mice [61] (Figure 1) (right).

As a novel molecular mechanism, we identified that *αB-crystallin* (*CRYAB*) is an important target gene of HSF2 and that induced CRYAB protein is indispensable for the suppression of polyQ protein aggregation [62]. CRYAB (another name is HSPB5) is a well-known small molecular chaperone. Intracellularly, it acts via its molecular chaperone action as the first line of defense in preventing the target protein from unfolding and aggregation under conditions of cellular stress. CRYAB can form large oligomeric complexes with itself and other small HSPs. CRYAB is highly expressed in the lens and to a lesser extent, in several other tissues, heart, skeletal muscle, and brain, and plays a role in several cellular processes such as signal transduction, protein degradation, stabilization of cytoskeletal structures, and apoptosis. Mutations in the *CRYAB* gene can have detrimental effects and lead to pathologies such as cataracts and cardiomyopathy [63,64].

Because CRYAB has various functions against misfolded protein aggregation, the HSF2-CRYAB pathway might be one of the main pathways in polyQ disease suppression; however, there will be unknown pathways and mechanisms through HSF2 activity. Sistonen and her colleagues discovered that HSF2 regulates cadherin superfamily genes and maintains protein homeostasis (proteostasis [65]) for cell survival against proteotoxicity including protein aggregation [66]. Subbarao and his colleagues suggested that HSF2 controls non-selective (micro/macro) and selective (chaperone-mediated) autophagy [67].

The fact that even *HSF2*-hetero HD mice showed a shortened life span and increased polyQ aggregation indicate that transcription of *HSF2* mRNA and synthesis of HSF2 protein may be more strictly regulated than we now suppose.

**Significance of Section 3:**

HSF2 is indispensable for the suppression of polyQ disease onset and progression.

HSF2 induced *αB-crystallin* (*CRYAB*) gene expression and suppresses polyQ disease.

The amount of mRNA and protein of HSF2 itself may be strictly regulated.

Other HSF2-mediated mechanisms have been reported in addition to the HSF2-CRYAB pathway; thus, several essential molecular mechanisms will contribute to the HSF2-mediated polyQ disease suppression.

## 4. HSF2 Has Essential Protective Roles in Ulcerative Colitis

Ulcerative colitis (UC) and Crohn’s disease (CD) are classified as chronic inflammatory bowel diseases (IBD) and lifelong illnesses which have a significant impact on quality of life. UC and CD have similar symptoms and lead to digestive disorders and inflammation in the digestive system [68,69]. Recent genome-wide association studies (GWAS) identified that *IL-12B* and *IL-23R* are susceptibility genes for inflammatory bowel disease (IBD) [70,71].

Brand and his colleagues analyzed *IL-12B* gene variants regarding association with UC and CD. At first, they analyzed four SNPs in the *IL12B* gene region in genomic DNA from 2196 individuals including 913 CD patients, 318 UC patients, and 965 healthy, unrelated controls, and found an association of the *IL-12B* SNP rs6887695 with susceptibility to IBD, CD, and UC [72]. Subsequently, Brand’s group performed in silico analysis and acquired the data predicting stronger binding of the major G allele of rs6887695 to HSF2 and HSF1. The binding scores of HSF2 and HSF1 to the minor C allele of re6887695 compared to the major G allele were decreased by 20.1% and 20.6%, respectively [72].

On the other hand, Miao and his colleagues discovered that HSF2 is prominently involved in UC development. In order to identify UC-associated proteins as biomarkers for the diagnosis, and objective assessment of disease activity, they examined serum proteins from UC patients and normal controls. They found that HSF2 expression in the colonic mucosa of UC subjects with varying severity of disease by real time-PCR and Western blot analysis. Moreover, the serum concentration of HSF2 positively correlated with the levels of IL-1β and TNF-α [73].

They more deeply studied the relationship between HSF2 and UC. They thought that mucosal healing is a treatment goal in UC, and examined whether HSF2 has mechanisms in the mucosal healing of UC. They collected fecal samples from 51 UC patients and 10 healthy controls, and performed a correlation analyses between HSF2 and fecal calprotectin (FC). The presence of FC is a consequence of neutrophil migration into the gastrointestinal tissue due to an inflammatory process. FC concentrations demonstrate good correlation with intestinal inflammation; therefore, it is used as a biomarker in gastrointestinal disorders [74]. They also transfected *HSF2* siRNA or *HSF2*-Flag recombinant plasmids into HT-29 colorectal adenocarcinoma cells, and measured the IL-1β, TNF-α, and TGF-β levels in supernatants by ELISA. In addition, they analyzed the molecular mechanism. These experiments showed that HSF2 decreased IL-1β and TNF-α secretion via suppression of the MAPK signaling pathway activation and that HSF2 promoted the expression of TGF-β via increasing phosphorylation of Smad2/3. As a result, they described that HSF2 may be a predictor of mucosal healing in UC patients and that HSF2 inhibits inflammation and promotes mucosal repair [75].

Miao’s group actively studied the functions of HSF2 in UC. They also performed in vivo experiments using *hsf2*-deficient mice because they hypothesized that HSF2 may protect against intestinal mucositis by regulating the apoptosis of intestinal epithelial cells (IECs). In this study, a DSS (dextran sulfate sodium)-induced colitis model of *hsf2*-deficient mice was used to explore the relationship between HSF2 and apoptosis in IECs. The expression of HSF2 increased in the WT + DSS group compared with that in the WT + H_2_O group, and the extent of apoptosis was more severe in the KO + DSS group than in the WT + DSS group. These results showed that HSF2 was negatively correlated with apoptosis in vivo.

They also changed the expression of HSF2 in Caco-2 colorectal adenocarcinoma cells by lentiviral transfection, and found that the expression of Bax, cytoplasmic Cyto-C, Cleaved Caspase-9, and Cleaved Caspase-3 were negatively correlated with the different levels of HSF2. From these results, they suggest that HSF2 negatively regulates the apoptosis of IECs through the mitochondrial pathway and that this may be one of the potential mechanisms to explain the protective role of HSF2 in UC [76].

The results indicated by the studies of Miao’s group are very interesting and may possibly lead to the development of novel therapy for UC. If their results are reproduced by other groups, these data must contribute to the progress of HSF2 research and discoveries of novel therapeutics for UC, IBD, and other inflammation diseases.

**Significance of Section 4:**

HSF2 is essential for the protection of ulcerative colitis (UC).

HSF2 inhibits the secretion of inflammatory cytokines and suppresses inflammation.

HSF2 inhibits apoptosis of intestinal epithelial cells and promotes mucosal repair.

## 5. HSF2 Affects Cancer Development and Progression

### 5.1. Involvement of HSF2 in Cancer Was Discovered Later Than HSF1

Compared to HSF2, it is now widely known that HSF1 and its target HSPs are positively related to cancer (tumor) development and progression [77,78,79]. The first evidence comes from studies on prostate cancer. We describe the short history in which HSF1 has been recognized to have strong and positive involvement in cancer before discussing novel discoveries in HSF2 and cancer relationship.

At first, Roy-Burman and his colleagues compared the non-metastatic human prostatic adenocarcinoma cell line PC-3 and the metastatic variant PC-3M, and discovered that HSF1 is expressed at a higher level in PC-3M than PC-3 in both mRNA and protein for the first time [80]. They also investigated the carcinomatous prostate tissue sections from patients and discovered that HSF1 protein was expressed more highly in tumor tissues than in normal sections from the same patient [80]. Subsequently, Calderwood and his colleagues noticed aneuploidy in PC-3 cells and thought that HSF1 probably has some role in this aneuploidy. They constructed a dominant-negative HSF1 (DN-HSF1, a mutant HSF1 lacking in transcriptional activity) expressing-vector, and established DN-HSF1 stably expressing PC-3 cells. They successfully discovered that aneuploidy was inhibited in these cells [81].

These data and results shown by these two groups had a high impact, and many papers indicating the relationship between HSF1 and cancer were published one after another. Among them, the paper published by Lindquist and her colleagues [82] showed the data by various experiments and have been cited by many papers. They performed both in vitro and in vivo experiments. In in vitro experimental data, they used various cancer cells including five human breast cancer cells and WI-38 normal fibroblasts. They showed that knockdown of HSF1 strongly inhibited the survival of various cancer cells, whereas WI-38 normal fibroblast cells were not affected [82].

They also showed that *HSF1*-deficient mice generated lesser number of tumors than wild-type mice in a skin cancer model and even that all the mice had mutations of the *Ras* oncogene or a hot spot mutation in the tumor suppressor *p53* gene.

### 5.2. Discovery of the Relationship between HSF2 and Various Cancers

#### 5.2.1. HSF2 Is Constitutively Active in Embryonic Carcinoma Cells

As described, the relationship between HSF1 and cancer has been studied since the first discovery; however, whether HSF2 affects cancer cell survival and other tumor characteristics had been not clearly shown. The first paper to indicate that HSF2 has some role in cancer is probably the report by Phillips and his colleagues [83] (Table 1).

The mouse teratocarcinoma (embryonal carcinoma) F9 cells (original F9 is a name of embryonic antigen) were frequently used for differentiation experiments in many laboratories as the multipotent cell line before ES cells can be easily used all over the world [84,85,86].

Phillips and his colleagues used F9 cells in this report, and they showed some clear data indicating that HSF2 is constitutively active in F9 cells under 37 degrees, forms dimer and trimer, and acquires DNA-binding activity under 37 degrees. HSF1 is not activated under 37 degrees and is activated under 43 degrees heat-stressed conditions in F9. However, HSF2 strangely did not bind to *HSP70* promoter and HSP70 protein expression was not observed [83]. As already described, overexpression of HSF2 can increase the expression of not only HSP70 but also HSP27, HSP47, and HSP90 in normal lung epithelial and lung cancer cells [57]. Phillips and his colleagues also suggested that the *HSP70* promoter was not opened due to the low expression level of HSF2. Similarly, as HSF2 is expressed at low level in mouse embryonic fibroblast (MEF) cells, *HSP70* mRNA may be not expressed [87].

We can understand that enough of an amount of HSF2 protein is probably required for the binding to *HSP70* promoter and *HSP70* induction under non heat-stressed conditions. In addition, in Huntington’s disease model (HD) mice, *WT*-HD, *HSF2*-hetero-HD, and *HSF2*-KO-HD mice showed different life spans [62]. The protein amount of HSF2 may be critically important for HSF2 to function compared to HSF1.

#### 5.2.2. HSF2 in Hepatocellular Carcinoma

The Wnt signaling pathway is involved in many differentiation events during embryonic development and can lead to tumor formation after aberrant activation of its components. Koman and his colleagues reported that HSF2 is a novel target of Wnt/β-catenin/TCF (T cell factor) signaling in hepatocellular carcinoma [88] (Table 1).

They used hepatocellular carcinoma-derived Huh7 cells; it is noteworthy that Wnt/β-catenin/TCF pathway is inactive and that endogenous β-catenin is not accumulated in the nucleus in Huh7 cells [89]. As Huh7 cells have this character, they used and transfected Huh7 cells with the vector-expressing mutant β-catenin with Ser33Tyr mutation, and established the TCF highly active Huh7 cells which have the character to lead more rapidly to larger tumors in nude mice when compared to the TCF less active Huh7 cells transfected with control vector. After they xenografted these two different cells to nude mice, they performed SAGE (Serial Analysis of Gene Expression), genome-wide microarray and in silico promoter analysis in parallel, and compared the gene expression between high and low β-catenin/TCF activity cells. They also examined the cells from the xenograft tumors and discovered that HSF2 is a novel target of Wnt/β-catenin/TCF signaling [88].

Meng and his colleagues reported that HSF2 regulate aerobic glycolysis in hepatocellular carcinoma through the suppression of fructose-1,6-biphosphatase 1 (FBP1) [90] (Table 1). FBP1 is a gluconeogenesis enzyme, and inhibits glycolysis and tumor growth, partly by non-enzymatic mechanisms [91]. Glycolysis is an indispensable mechanism for tumor cells to survive; thus, gluconeogenesis function of FBP1 consequently threatens tumor cell survival. In fact, downregulation of FBP1 was discovered in gastric carcinomas and gastric cancer cell lines [92] and FBP1 was found to inhibit pancreatic cancer progression [93].

To date, several mechanisms for how HSF2 affects the characteristics of hepatocellular carcinoma have been suggested. As described, one of them is that HSF2 contributes to the hepatocellular carcinoma survival as a part of the Wnt/β-catenin/TCF pathway, and the other one is that HSF2 suppresses FBP1 and upregulates aerobic glycolysis essential for hepatocellular carcinoma growth.

Recently, Guo and his colleagues showed that high expression of DNAJC24 (HSP40 Member C24, a heat shock protein) occurs in hepatocellular carcinoma patients with shortened life span and that the transcription of DNAJC24 is regulated by HSF2 [94] (Table 1).

They examined a total of 167 pairs of cancerous and nonmalignant tissue samples obtained from the same patients with hepatocellular carcinoma by immunohistochemistry staining for DNAJC24. High expression of DNAJC24 was observed in hepatocellular carcinoma cells but not in nonmalignant cells. Next, they performed the correlation analysis using the Cancer Genome Atlas (TCGA) database and determined that HSF2 expression is positively correlated with DNAJC24 higher than other HSFs. Additionally, HSF2 expression was higher in hepatocellular carcinoma tissues than in para-cancerous tissues, and the HSF2 expression level increased with tumor stage [94]. These data indicate that the HSF2-DNAJC24 pathway is also involved in the HSF2-mediated mechanism of hepatocellular carcinoma survival or progression.

Other novel mechanisms related to HSF2 must be continuously discovered. In not only hepatocellular carcinoma but also other carcinomas, HSF2 controls a few or several pathways at the same time and helps tumor growth. We will need to search chemicals modulating the function or expression of HSF2 and find that a few best ones and test them in cancer therapy as soon as possible.

#### 5.2.3. HSF2 in Head and Neck Squamous Cell Carcinoma

Steinhart and his colleagues tried to acquire information on the antigenic profile of head and neck squamous cell carcinomas (HNSCC) and performed serological analysis of tumor antigens by recombinant cDNA expression cloning (SEREX). They found that antibodies against HSF2 exist in 2 out of 10 sera from HNSCC patients [95] (Table 1). These data indicate the possibility that HSF2 is an antigenic protein of HNSCC and may be a target for an immunotherapy approach.

#### 5.2.4. HSF2 in Breast Cancer

Zhu and his colleagues investigated the regulation mechanisms of the *miR-183/-96/-182* cluster in breast cancer because this cluster is a conserved polycistronic microRNA (miRNA) cluster which is highly expressed in most breast cancers. They found that this *miR-183/-96/-182* cluster was transcribed in the same pri-miRNA and discovered that its transcription was regulated by HSF2 [96] (Table 1).

In addition to HSF2- *miR-183/-96/-182* cluster pathway, HSF2-ALG3 pathway was discovered in breast cancer growth mechanism [97] (Table 1). ALG3 (*alg3*, asparagine-linked glycosylation 3) is a gene originally found in yeast *Saccharomyces cerevisiae*, and encodes mannosyltransferase required for oligosaccharide-lipid elongation [98]. In general, cancer cells have abnormal oligosaccharide chains; thus, involvement of ALG3 was reported in squamous cell cervical cancer, acute myeloid leukemia (AML), head and neck squamous cell carcinoma, non-small cell lung cancer, and oral squamous cell carcinoma [99,100,101,102,103]. There is a strong possibility that the HSF2-ALG3 pathway will be discovered in other cancers.

#### 5.2.5. HSF2 in Lung Cancer

Wang and his colleagues examined lung cancer tissues, and discovered that the expression level of *HSF2* mRNA is significantly upregulated in lung cancer tissues compared to normal tissues [57] (Table 1). As well as mRNA, HSF2 protein was also increased.

As already described in Section 2 of this paper, they also showed that overexpression of HSF2 by transfection increased the expression of HSP27, HSP47, HSP70, and HSP90 proteins in both normal lung epithelial and lung cancer cells. Many papers have reported the increased expression of HSPs in cancer including lung cancer [104,105,106]; these increased expressions of HSPs were already reported in culture cell experiments in the 1980s [107,108] and discovered in the specimen from patients in the 1990s [109,110]. These phenomena were observed earlier than the discovery of the relationship between cancer and HSF1 in 2000.

The relationship between cancer and HSF2 was discovered in this decade. However, unexpectedly, only two papers reported that HSF2 has a relationship with lung cancer. In addition, one paper did not show important data about HSF2, and the other paper by Wang and his colleagues is the only one substantially reporting the involvement of HSF2 in lung cancer [57]. There may be a non HSF2-mediated cancerous pathway or mechanism as HSF2 protein is expressed enough in lungs; therefore, there must be a HSF2-mediated essential mechanism in lung cancer generation or progression. To treat lung cancer patients, we must investigate and find drugs and therapeutics controlling HSF2 activity.

#### 5.2.6. HSF2 in Thyroid Cancer

Zhang and his colleagues tried to reveal the molecular mechanism of thyroid carcinoma. They collected RNA-sequencing data of thyroid carcinoma (N = 498) and normal thyroid (N = 59) tissues (these data were downloaded from The Cancer Genome Atlas) and examined it using the bioinformatics technique. They identified 254 upregulated and 59 downregulated differentially expressed genes (DEGs) of which expression levels were different between carcinoma and normal tissues [111]. In addition, they examined what molecules regulate the expression of DEGs, and identified that five miRNAs and five transcription factors including HSF2 regulate DEGs. Through this analysis, they thought that HSF2 is involved in thyroid carcinoma development by regulating *SERPINA1* and *FosB* genes (Table 1).

*SERPINA1* (Serpin family A member 1) encodes a well-known elastase inhibitor, alpha-1 antitrypsin (AAT). Previously, Wiseman and his colleagues reported that SERPINA1 can be diagnostic biomarker of thyroid cancer [112], and SERPINA1 has been recently reported to be involved in several other cancers including gastric, liver, pancreas, breast, lung, and ovarian cancers [113,114,115,116,117,118]. Moreover, Gao and his colleagues suggested that SERPINA1 is one of key genes in brain metastasis from lung adenocarcinoma [119].

Zhang and his colleagues also described that HSF2 may be involved in thyroid carcinoma development by regulating *FosB* [111], Fos is the one of transcription factor families and this Fos family includes c-Fos (the human homologue of retroviral oncogene v-Fos), FosB, Fra-1 and Fra-2 as well as smaller FosB splicing variants FosB2 and deltaFosB2 [120]. Fos (c-Fos, FosB, Fra1, and Fra2) and Jun (c-Jun, JunB, and JunD) families form AP-1 dimeric transcription factor, AP-1 is activated by various stimuli such as inflammatory cytokines, stress inducers, or pathogens, and is involved in cell differentiation, proliferation, survival, and death (apoptosis). It is known that AP-1 is involved in various lymphomas such as classical Hodgkin lymphomas, anaplastic large cell lymphomas, diffuse large B-cell lymphomas, and adult T-cell leukemia [121,122].

Zhang and his colleagues showed that HSF2 regulates *FosB* in thyroid carcinoma development. HSF2 may contribute to carcinogenesis in the thyroid by activating the FosB-containing transcriptional complex such as the AP-1 or AP-1 complex.

#### 5.2.7. HSF2 in Esophageal Cancer

Esophageal cancer is one of the most common malignant cancers and the sixth most common cause of cancer-related death worldwide. There are two major histologic types, squamous cell carcinoma (ESCC) and adenocarcinoma (EAC). They are epidemiologically and biologically distinct. ESCC accounts for approximately 90% of all esophageal cancer patients globally and is highly prevalent in the East, East Africa, and South America. EAC is more common in developed countries than in developing countries [123,124,125].

Fan and his colleagues considered that an altered expression of miRNAs has been found in patients with different cancers, and that this has been associated with the pathogenesis of human cancers [126]. They examined miRNAs and reported the downregulation of miR-202 in ESCC for the first time [127]. Next, they searched the TargetScan and miRDB databases, explored the underlying mechanism of miR-202 action, and predicted the potential target of miR-202 in ESCC. Finally, they found that there is a possible binding site for miR-202 in the 3′-UTR of *HSF2* mRNA [124] (Table 1).

They also discovered that overexpression of miR-202 significantly reduces the HSF2 protein and that miR-202 inhibitor increases HSF2 protein. In addition, *HSF2* silencing by siRNA increased the rate of apoptosis and overexpression of HSF2 repressed this apoptosis by the induction of HSP70 and preventing caspase-3 activation [124].

To date, only Fan and his colleagues reported that HSF2 is positively involved in esophageal squamous cell carcinogenesis; however, aberrant regulation of miR-202 was also reported in human osteosarcoma, colorectal carcinoma, lung cancer, pancreatic cancer, bladder cancer, and prostate cancer [128,129,130,131,132,133]. As the decrease in miR-202 can directly upregulate HSF2, HSF2-miR-202 pathway will exist in various cancers and carcinogenesis.

#### 5.2.8. HSF2 in Prostate Cancer

As already described, the involvement of HSF in cancer was discovered in prostate carcinoma cells and tissues from prostate cancer patients for the first time in 2000 [80]. In this first report, published in 2000, Roy-Burman and his colleagues compared the non-metastatic human prostate carcinoma PC-3 and the metastatic variant PC-3M cells, and discovered that HSF1 is expressed at higher level in PC-3M than PC-3 in both mRNA and protein [80]. In the same report, they also discovered and showed that HSF1 protein was expressed more highly in tumor tissues than in normal sections from the same patient [80].

In 2016, although HSF1 has been well recognized as a driver of carcinogenesis, the involvement of HSF2 in various cancers had not yet been explored. Under these circumstances, Nees and Sistonen’s group reported that HSF2 suppresses tumor invasion of prostate cancer [134] (Table 1).

At first, they used the data obtained from the cBioPortal for Cancer Genomics (http://www.cbioportal.org/public-portal/) [135] and found that mRNA expression of HSF2 is highest in normal prostate tissue and is lower proportional to Gleason score (Gleason score is widely accepted as the standard for histologic grading of prostate malignancies [136]). In addition, they found that the homozygous deletion of HSF2 occurred in 18% of prostate tumor samples. In the same samples, mRNA upregulation of HSF1 occurred in 14% [134] (Table 1).

We will need to keep in mind the data shown in this report. In the cell culture experiments, the authors performed a 3D organotypic culture in the Matrigel and examined the mRNA expression, protein expression, and invasive behavior of several prostate cancer cell organoids. In these culture experiments, mRNA expression of *HSF2* were highest in non-invasive cells (LNCaP and Du145), second highest in invasive cells (PC-3 and PC-3M), and lowest in normal cells (PrEC and EP156T). Similar result was also obtained in Western blot analysis. EP156T (normal), luminal and non-invasive (LAPC4), and luminal and invasive (PC-3) cells were examined, luminal and non-invasive LAPC4 cells showed prominent high expression. Luminal and invasive PC-3 cells showed obvious expression, but normal EP156T cells only showed faint expression compared to LAPC4 and PC-3 cancer cells [134]. Moreover, knockdown of *HSF2* in PC-3 cells showed increased invasive ability, and overexpression of HSF2 in these *HSF2* knockdown cells showed the suppression of invasiveness.

Before their report in 2016, only one paper by Koman and his colleagues suggested that HSF2 may be positively involved in carcinogenesis using hepatocellular carcinoma [88]. In addition, as Wnt/β-catenin/TCF signaling has been known to cause tumor formation with aberrant activation of the components in this signaling, and they also showed that HSF2 is targeted by Wnt/β-catenin/TCF signaling and contributes to tumor formation [88].

As it happens, the publication of many papers demonstrating that HSF2 is involved in carcinoma and tumor formation has increased since the year 2016, almost all of them indicating that HSF2 is positively involved in cancer cell growth, survival, proliferation, and tumor formation; however, no other papers referring to the relationship between HSF2 and prostate cancer have been published since the report of Nees and Sistonen group.

However, we already know that biological phenomena appear in a different way due to the materials and methodology of cell culture or cell species. For example, HSPs suppress HSF1 activation, but Lee and his colleagues reported that HSP25 (HSP27) and HSP70 induce HSF1 activation in some sarcoma cells [137]. In response to heat stress, HSF1 is activated and induces Hsp70 and Hsp25 gene expression, but it is widely recognized that induced HSPs return HSF1 to its inactive monomeric state interacting with some HSPs in a negative feedback mechanism [138,139].

Since the discovery of heat shock response, we have accumulated a great deal of data, information, and knowledge. However, through the evolution and improvement of experimental methods, we will face strange but reproducible data from now on. Considering the broad functions of HSF2, we will not need to reach the conclusion in haste.

### 5.3. Other HSF2-Mediated Novel Mechanisms in Cancer Cells and Possibility to Discover Therapeutic Target

As described above, HSF2 has been revealed to be deeply involved and/or to have indispensable roles in many cancers. Then, how wide is the range of molecular mechanisms where HSF2 participates?

Recently, Mendillo and his colleagues reported that HSF2 physically and functionally interacts with HSF1 across diverse types of cancer [140]. It is widely known that HSF2 interacts with HSF1 [141,142], surprisingly, they performed The Cancer Genome Atlas (TCGA) gene expression analysis in 21 cancers and examined which HSF has highest expression. They also established four cancer cell lines with stably expressed HSF1 tagged with Flag, and decided that HSF2 is a highest-confidence HSF1-interacting protein [140].

Subsequently, they examined chromatin occupancy, and found that HSF2 and HSF1 have similar occupancy and regulate common sets of genes. HSPs were expectedly included in these common sets, but importantly, non-canonical target genes in these sets commonly have tumor malignancy supporting roles. Actually, loss of either HSF1 or HSF2 in cell experiments showed that these cells result in a dysregulated response to nutrient stresses in vitro and that tumor progression was reduced in cancer cell line xenografts.

Their report strongly indicates that the role of HSF2 in cancer biology is far greater than previously appreciated. As they described, HSF2 is probably an important partner (they expressed it as ‘critical accomplice’) of HSF1 in driving pro-tumorigenic gene expression programs [140].

In this section, we showed that many cancers are activated by HSF2. We mainly referred to eight cancers by making one part for one kind of cancer, but in addition, some papers report that HSF2 also has a relationship with (muscle invasive) bladder cancer and cervical cancer [143,144]. To date, new findings have not been reported in using *HSF2*-deicient mice. Lindquist and her colleagues used a classical multistep chemical skin carcinogenesis protocol and showed that striking difference in carcinogen-induced tumorigenesis occurred between *HSF1*^+/+^ mice and *HSF1*^−/−^ mice [82]. In these mice, somatic mutations were induced in epidermal cells by a single topical application of the mutagen dimethylbenzanthracene (DMBA). Then, tumor promotion is achieved by repeated applications of the phorbol ester 12-O-tetradecanoylphorbol-13-acetate (TPA). As a result, it was discovered that *HSF1*^−/−^ mice were far more resistant to tumor formation than *HSF1*^+/+^ mice. If similar results are obtained using *HSF2*^+/+^ and *HSF2*^−/−^ mice, the research on HSF2 in cancer will attract the attention of many more scientists.

The evidence that increased expression of both HSF1 and HSF2 have critically important roles in cancer development and progression has been accumulating year after year. If more detailed molecular mechanisms become clear, we may be able to proceed to the step of therapeutic development. At the same time, HSF2 has broader functions and roles in biological phenomena; therefore, the analysis of chemicals interacting with and/or affecting HSF2 must be performed with enough care to avoid the induction of side effects.

**Table 1:** List of studies on the relationship between HSF2 and cancers.

Type of Cancer	Report	Samples	Reference
Embryonic Carcinoma	HSF2 is constitutively active under 37 degrees condition	cell line	[83]
Hepatocellular Carcinoma	HSF2 is a novel target of Wnt/β-catenin/TCF signaling	cell line, xenograft	[88]
Hepatocellular Carcinoma	HSF2 regulates aerobic glycolysis through the suppression of FBP1	cell line	[90]
Hepatocellular Carcinoma	HSF2 regulates transcription of *DNAJC24*	patients	[94]
Head and Neck Squamous Cell Carcinoma	Anti-HSF2 antibodies are detected	patients	[95]
Breast Cancer	HSF2 regulates transcription of miR-183/-96/-182 cluster	patients and cell lines	[96]
Breast Cancer	HSF2 regulates transcription of *ALG3*	patients	[97]
Lung Cancer	Expression of HSF2 is significantly upregulated the expression of HSPs	cell line	[57]
Thyroid Carcinoma	HSF2 regulates transcription of *SERPINA1* and *FosB* genes	patients	[111]
Esophageal Squamous Cell Carcinoma	Expression of HSF2 is down regulated by miR-202 via binding site in the 3′-UTR of HSF2 mRNA	cell line	[124]
Prostate Cancer	Homozygous deletion of HSF2 was occurred in 18% of patient samples	patients	[134]
Prostatic Adenocarcinoma	mRNA expression of HSF2 in non-invasive cells were higher than invasive cells	cell lines	[134]

**Significance of Section 5:**

HSF2 is involved in 10 or more kinds of cancers (carcinogenesis and/or progression).

The relationship between HSF2 and cancer was reported in 2010 for the first time, but rapidly increased in the last 5–6 years.

Cancer experiments using *HSF2*^+/+^ and *HSF2*^−/−^ mice have not been performed or reported yet. If highly impactful data are obtained from the experiments using *HSF2*^+/+^ and *HSF2*^−/−^ mice, it must be a significant progress in both HSF2 and cancer research fields.

## 6. Mutation in Human *HSF2* Gene Causes Male Infertility

### 6.1. Discovery of Spermatogenesis Defects in HSF2-Deficient Male Mice

Recent novel findings related to the roles of HSF2 in normal fertility were summarized in our review [6]. In this previous review, we especially focused on meiosis and referred to both HSF2 and HSF1 because HSF1 has specific roles in the gene silencing and segregation of X- and Y- chromosomes [145,146]. Normal meiosis is indispensable for sexual reproduction; thus, meiotic failure seriously affects the production of offspring. Meiosis produces haploid cells from diploid parental cells and reduces the chromosome number by half. The regulation mechanisms of the cell cycle in meiosis are different from those in mitosis, and the disturbance in meiosis occurring in spermatogenesis and oogenesis affects the production of normal sperms and oocytes as described [6]. In 2002 and 2003, three different groups reported the phenotypes of their original *HSF2*-deficient mice [27,28,147]. These studies revealed that HSF2 is required for normal fertility for the first time. We want to refer to their novel discoveries related to spermatogenesis here.

Mezger and her colleague investigated testes and sperms in detail. Testes in *HSF2*^−/−^ mice were significantly smaller than in *HSF2*^+/+^ mice. The average diameter of seminiferous tubules in the *HSF2*^−/−^ testis was also significantly smaller than in *HSF2*^+/+^ mice. In the tubules of *HSF2*^+/+^ mice, normal amounts of differentiating germ cells from different developmental steps organized into the typical layer pattern [27]. However, there was disruption of spermatogenesis such as degenerating cells with condensed nuclei, lack of certain differentiating spermatocytes and spermatids, and vacuolization in many of the tubules of *HSF2*^−/−^ mice. The number of sperm found in the epidermis of the *HSF2*^−/−^ mice was significantly lower in comparison with the number of sperm of *HSF2*^+/+^ mice. In addition, apoptotic cells were increased in *HSF2*^−/−^ mouse testes about three times more than in *HSF2*^+/+^ testes.

Mezger and her colleagues described more novel findings in a 2002 paper, observing that many developing spermatocytes died and disappeared by apoptosis in a stage-specific manner (they judged this from the fact that the dying cells in the *HSF2*^−/−^ mouse testes were always found in clusters). Structural defects were also observed in pachytene spermatocytes in the synaptonemal complexes between homologous chromosomes in *HSF2*-deficient male mice.

Benjamin and his colleagues hypothesized that HSF2 may play an essential role in brain and heart development, spermatogenesis, and erythroid differentiation; thus, established their original *HSF2*^−/−^ mice [147]. Unexpectedly, their *HSF2*^−/−^ mice are viable and fertile and exhibit a normal life span and behavioral functions. Mivechi and her colleagues also established their original *HSF2*^−/−^ mice and analyzed them, they reported various phenotypes [28]. Their *HSF2*^−/−^ mice showed an increased prenatal lethality occurring between mid-gestation and birth, with fetal death probably due to central nervous system defects including collapse of the lateral ventricles and ventricular hemorrhages. Concerning spermatogenesis, they described that in markedly reduced testis size and sperm count, only a small reduction in fertility was apparent in their *HSF2*^−/−^ male mice [28].

These three groups used a different protocol, but they all showed the data on the successful establishment of their *HSF2*^−/−^ mice by Southern blot, RT-PCR, and other data. There are no doubts about the establishment, but we noticed that the HSF2 band exists in the kidney in the Northern blot data of *HSF2*^+/+^ (no band exists in *HSF2*^−/−^ mice) by the Benjamin group [147]. Brown and his colleagues examined the expression of HSF2 in rat brain and kidneys and showed that HSF2 protein expression completely disappear in kidneys postnatally at 30–98 days [148]. We also have examined and confirmed that the HSF2 protein was not expressed in kidneys in normal C57BL/6 mice. Although Brown group examined rats, not mice, it seems a little strange for HSF2 to express in the kidney.

Now, almost all establishment of genetic mutant mice has been performed using the Crispr-Cas9 technique. This technique is fast and made easy to obtain in the mice; however, unexpected mutation frequently inserts and thus the confirmation of the recombinant sequence must be performed. Three *HSF2*^−/−^ mice were established by the homologous recombination technique in ES cells, but re-confirmation of the DNA sequence of these mice might be performed again for HSF2 research progress in the 21st century.

### 6.2. Discovery of Azoospermic Patients with HSF2 Mutation

Azoospermia may occur because of reproductive tract obstruction (obstructive azoospermia, OA) or inadequate production of spermatozoa, such that spermatozoa do not appear in the ejaculate (non-obstructive azoospermia, NOA) [149]. Azoospermia is identified in 1% of all men and up to 20% of infertile men. One is subdivided as OA and the other is as NOA [150,151].

In 15% of couples who wish to have children, about half of these cases are thought to be associated with male factors. NOA is one of the most severe forms of male infertility because of impaired spermatogenesis with the absence of spermatozoa in the ejaculate [152]. For men with OA, several surgical sperm retrieval techniques can facilitate conception with assisted reproductive technology [153].

The first azoospermic patients (patients with infertility) with HSF2 loss-of-function mutation have been discovered by Gui and his colleagues in 2013 [154]. As they described, they knew that *HSF2*-deficient male mice showed abnormal spermatogenesis; therefore, they examined the *HSF2* gene sequence. They described ‘Idiopathic azoospermia (IA) is a severe form of male infertility due to unknown causes. The *HSF2* gene, encoding the heat shock transcription factor 2, had been suggested to play a significant role in the spermatogenesis process since the *Hsf2*-knockout male mice showed spermatogenesis defects. To examine whether HSF2 is involved in the pathogenesis of IA in human, we sequenced all the exons of HSF2 in 766 patients diagnosed with IA and 521 proven fertile men’ in their abstract [154].

Needless to say, the information brought by the analysis of their *HSF2*-deficient mice independently established by Mezger’s group, Benjamin’s group, and Mivechi’s group made it possible for Gui’s group to discover that the HSF2 loss-of-function mutation causes human male infertility for the first time. The contribution of these three groups should be appreciated.

Gui and his colleagues sequenced all the exons of *HSF2* (on chromosome 6) in 766 patients diagnosed with IA and 521 proven fertile men. IA is a severe azoospermia of male infertility because it is very difficult to uncover what causes IA. They recruited these patients from January 2007 to October 2011 [154]. Genomic DNA (5 μg per one patient) was isolated from the peripheral blood samples.

By the sequencing of all exons of *HSF2*, a number of coding mutations were discovered in the patient group. These include nine synonymous mutations and five missense mutations. Furthermore, this group identified one characteristic mutation from five missense ones. They focused on this mutation, and subsequently performed several functional assays, and identified that this is one heterozygous mutation resulting in Arg502His amino acid replacement [154].

Arg residue at the 502 position is very conserved at least among nine species including human, mouse, cow, chicken, and zebrafish. In most cases, highly conserved amino acid is indispensable for the function and/or activity of the protein; thus, they next performed a luciferase assay using the promoter sequence of *HSPA2* (testicular *HSP70*), a well-known target gene of HSF2 in sperm.

This mutation (replacement) caused complete loss of HSF2 function, and in addition, they found that this mutant has a dominant-negative effect; therefore, this Arg502His mutant HSF2 suppressed the function of normal HSF2 encoded by the wild-type allele.

The research of Gui and his colleagues showed that the HSF2 mutation causes male infertility for the first time. It is still important to discover the mutations in the *HSF2* gene involved in infertility, but it must also be important to reveal the mechanism of how Arg502His mutant HSF2 loses its functions completely. Arg502 positions in the activation domain Y of human HSF2; however, what conformation this domain takes and what other molecules interact with this activation we do not have enough information on. Both Arg and His have a positive charge; it is speculated that the change in structure around Arg502 should be destroyed by the replacement to His. His has a characteristic large five-membered ring.

In 2020, Liu and his colleagues reported that novel *HSF2* mutation causes infertility [152]. They examined a Chinese infertile 27-year-old man. This patient showed a sperm concentration of 0/mL in 6.5 mL of semen. After performing the other comprehensive examinations, the patient was diagnosed with NOA.

They isolated genomic DNA of this patient from blood lymphocytes and performed sequencing analysis of spermatogenesis associated genes. Liu and his colleagues discovered a novel deletion–insertion variation c.326_326delinsGGAAGGTGAGCTATTGT in the exon 3 of the *HSF2* gene. They also performed Sanger sequencing and confirmed the heterozygous missing insertion mutation in this patient [152].

Subsequently, they used The National Center for Biotechnology Information (NCBI) database and predicted the transcription of this mutated *HSF2* gene. They found that the length of amino acid sequence was shortened to 124aa, and transcription was terminated prematurely. Among the 124 amino acids transcribed, change in amino acids occurred from the 113th Serine. The 113th amino acid was changed from Serine (Ser) to Tyrosine (Tyr). The 113–124 amino acid sequence is Tyr-Cys-Glu-Gly-Phe-Ile-Phe-Lys-Tyr-Arg-Arg-Lys (YCEGFIFKTRRK). The last amino acid is Lysine (Lys) [152]. If this transcript is normally translated as well as other transcripts, finally synthesized mutant HSF2 proteins have almost only a DNA-binding domain (DBD). In many transcription factors, translated peptides corresponding to DBD are folded to the DBD structure without difficulties. This is our hypothesis, that this mutant HSF2 will be synthesized and folded to the small protein with DBD activity. Furthermore, this mutant HSF2 will be able to prevent the binding to HSE sequence of other HSFs and normal HSF2. It is possible for this protein to have several functions.

**Significance of Section 6:**

HSF2 mutations causing male infertility were discovered.

The *HSF2* gene mutation discovered to cause male infertility for the first time is loss-of-function mutation.

Establishment of *HSF2*^−/−^ mice and discovery of abnormalities in spermatogenesis in the *HSF2*^−/−^ male mice gave researchers the opportunity to analyze the sequence of the *HSF2* gene in the first azoospermic patient.

The first discovered *HSF2* mutation caused Arginine (Arg) to Histidine (His) amino acid replacement at the 502 position in HSF2 activation domain.

The second mutation reported in 2020 is c.326_326delinsGGAAGGTGAGCTATTGT in the exon 3 of the *HSF2* gene.

The mutant *HSF2* produced by novel deletion–insertion variation has only 124 amino acids and most part is DNA-binding domain.

Both mutant HSF2s may prevent the binding to the HSE sequence of normal HSF2 and other HSFs.

## 7. HSF2 Is an Essential Mediator in Fetal Alcohol Spectrum Disorder (FASD) and a Critical Response Factor to Ethanol-Induced Stress

### 7.1. Maternal Consumption of Alcohol during Pregnancy Causes Fetal Alcohol Spectrum Disorder (FASD) and Fetal Alcohol Syndrome (FAS)

Several diseases of the central nervous system (CNS), early stages of these diseases probably begin during the early development of CNS [155]. Various factors can alter the normal development of CNS during gestation.

Alcohol equilibrates freely from maternal to fetal circulation, disrupting maternal, placental, and fetal physiology in both animals and humans, and prenatal exposure to alcohol can affect fetal brain development at any point of gestation [156,157].

Fetal alcohol spectrum disorders (FASD) are caused by the maternal consumption of alcohol during pregnancy. According to the Updated Clinical Guidelines for Diagnosing FASD 2016, FASD can result from prenatal alcohol exposure and comprises a range of symptoms, including minor craniofacial anomalies, growth retardation, neurological abnormalities, cognitive and behavioral impairment, and birth defects [158,159]. In addition, the most severe clinical manifestations, referred to as fetal alcohol syndrome (FAS), include dysmorphic facial features, intrauterine growth defects, and brain lesions [160,161,162]. Postmortem brains of FAS infants show reduced brain weight, disturbance in horizontal cortical lamination, neuronal ectopias, and/or a reduced thickness of the cortical mantle [156,161,162,163].

Ethanol (EtOH)-induced cell death during embryogenesis has been examined by many researchers. Among them, Sulik and his colleagues examined the patterns of both normal apoptosis and EtOH-induced cell death in the CNS and craniofacial region at 0.5-day intervals from 6.5 to 11 gestational days (GD) in mice in detail [164].

They described the regions with normal programmed cell death (apoptosis) in control embryos (without EtOH injection) as follows:

GD 8.0; prechordal plate region

GD 9.0; the neuroepithelium of the fourth ventricle and anterior neuropore

GD 10.0; the ganglia of cranial nerves V, VII-VIII, IX, and X

EtOH-induced apoptosis was described as follows:

GD 7.5; neuroectoderm

GD 8.0; neural plate and primitive streak

GD 9.0; alar plate and presumptive neural crest of the rostral hindbrain, especially at the mesencephalon/rhombencephalon junction

GD 9.5–10.0; branchial arches and rhombomeres

GD 11.0; diencephalon, basal ganglia, pons, and developing cerebellum

In the control embryos, they could specifically describe the region; however, they described only the regions because maternal EtOH administration induced a prominent increase in apoptosis in the embryos. Moreover, EtOH-exposed specimens exhibited stage-dependent excessive cell death in other distinct cell populations, particularly within the developing central nervous system [164].

From these results, Sulik and his colleagues concluded that the developmental stage-specific cell populations of the developing brain and craniofacial region are vulnerable to EtOH-induced apoptosis [164]. The difference of vulnerability of specific cell populations of the developing brain to EtOH-induced apoptosis may explain how maternal EtOH consumption causes FASD-specific symptoms and defects.

### 7.2. HSF2 Is a Master HSF Working during Embryo Growth and Its Deficiency Causes Abnormalities in CNS Development

During development, one-cell and two-cell embryos respond to heat stress and strong HSE-binding activity can be detected [165]. At the four-cell stage, heat shock-induced HSE-binding activity eliminates, but reappears between the eight-cell stage and the blastocyst stage. Moreover, HSE-binding activity at normal temperature can be observed at the morula stage and increased at the blastocyst stage [165].

As described above, Mezger and her co-researchers found the presence of an abundant HSE-binding activity in non-shocked blastocysts at room temperature. Different from HSF1, HSF2 is active at normal temperatures. HSF2 is the major constituent of this constitutive HSE-binding activity during development [166].

Mezger and her colleagues subsequently established *HSF2*-deficient mice. These mice showed abnormal structure of adult brain without any stresses during development. Lateral and third ventricles all along the brain was enlarged, and hippocampus and striatum sizes were reduced [27]. They also found that HSF2 is only expressed in proliferative cells of the neuroepithelium and in some ependymal layer cells near the ventricular zone in adult brain. Mivechi and her colleagues also established their original *HSF2*-deficient mice and analyzed phenotypes [28]. They discovered CNS defects, and these defects include collapse of the lateral ventricles and ventricular hemorrhages. Approximately 30% of *HSF2*-deficient mice growing to adulthood showed marked dilation of the third and lateral ventricles. It is noteworthy that these two lines of *HSF2*-deficient mice established by different groups commonly showed CNS defects especially in the third and lateral ventricles.

As already shown, Sulik and his colleagues reported that apoptosis is induced in CNS during normal development. Apoptotic cells are observed in the prechordal plate region (at GD 8.0), neuroepithelium of the fourth ventricle and anterior neuropore (at GD 9.0), and ganglia of cranial nerves V, VII–VIII, IX, and X (at GD 10.0) [164]. Comparing the data shown in the papers from Sulik, Mezger, and Mivechi groups, it seems that the apoptotic cell appearance area coincides with the characteristic phenotypes observed area in the *HSF2*-deficient mice, not completely but partially at least.

### 7.3. HSF2 Is Involved in FASD and Its Deficiency Mitigates the Influence of EtOH Exposure

In 2014, Mezger and her colleagues reported that HSF2 is directly involved in FASD. They used their *HSF2*-deficient mice and cell culture systems, and found that EtOH exposure elicited more prominent neuronal cell positioning abnormalities in WT-mice than *HSF2*-deficient mice [162].

This result seems to be unexpected and strange because HSF2 acts for maintaining and protecting normal conditions of tissues and organs including brain in most of cases, for example, in neurodegenerative diseases such as polyQ diseases (see Section 3). However, Mezger and her colleagues examined this mechanism and proved the correctness of their results.

In previous studies, they showed that HSF2 influences brain cortical development through regulating *non-HSP* genes [24,27,28]. As already known, post-mitotic neurons are generated from dividing neuronal progenitors in the inner parts of the developing cortex (the ventricular zone, VZ), and next migrate from VZ radically to more external positions during mammalian corticogenesis. Finally, radial neuronal migration forms six-layer cortex [167]. Mezger’s group previously revealed that HSF2 influences radial neuronal migration by regulating the expression of p35 and p39. Of these genes, HSF2 directly bound to the *p35* promoter (HSE exists from −66 to −43) in E16.5 cortical tissue [29]. p35 and p39 proteins activate cyclin-dependent kinase 5 (Cdk5), and this mechanism is required for neuronal migration [29]. However, the expression level of both p35 and p39 were decreased in *HSF2*-deficient cortices and Cdk activity of Cdk5 was also reduced [29]. It is well known that a lot of genes are involved in radial neuronal migration, for example, some microtubule-associated proteins (MAPs) such as Doublecortin (Dcx) and Doublecortin-like kinase 1 (Dclk1) are famous to have critical roles in corticogenesis [168,169,170,171].

Under these circumstances, Mezger and her colleagues observed the novel important phenomenon. They discovered a novel mechanism that HSF2 controls Cdk5 activity through the regulation of p35 and p39, but chronic prenatal alcohol exposure in the cortex prominently disturbed this mechanism [162]. In detail, they gave alcohol (EtOH)-containing food (CAI) to pregnant female mice. Under control conditions, in the pregnant mice eating only normal food, HSF2 is constitutively active but HSF1 is mostly inactive. In contrast, CAI induced HSF1 activation and increased the nuclear localization of HSF1 in the cortical plate and HSF1 phosphorylation on Ser residue at position 326. Phosphorylation at 326 means activation of HSF1 [172,173]. In addition, CAI maintained HSF2 activation; however, both mRNA and protein expression level of p35, Dcx, and Dclk1 was significantly reduced (but HSP70 was increased) and the binding of HSF2 to the promoters of these genes was also decreased. These data suggest that alcohol (EtOH) consumption disturbs the cortex development mechanism at least by reducing the expression of p35, Dcx, and Dclk1.

### 7.4. HSF2 Is Involved in Synaptic Plasticity Disturbance Caused by EtOH Consumption

Synaptic plasticity plays an important role in learning and memory. The discovery of long-term potentiation (LTP) and long-term depression (LTD) is critically important, and these two phenomena most widely used as synaptic plasticity model in many experiments [174].

In the CNS, the main neurotransmitter is glutamate (Glu); thus, several ionotropic Glu receptors exist in the surface membrane of postsynaptic neurons. Their primary function is to respond to pre-synaptically released neurotransmitter by generating depolarization (this means ‘excitation’) and developing excitatory postsynaptic potentials (EPSPs) [175].

The majority of EPSPs in the mammalian CNS are mediated by well-known subtypes of ion channel Glu receptors, α-amino-3-hydroxy-5-isoxazole-propionic acid (AMPA), and *N*-methyl-D-aspartic acid (NMDA) receptors [176]. NMDA receptors consist of four major subunits, in particular, the combination of two GluN2A and two GluN2B receptors is a major subunit formation [177].

The major form of LTP observed in the brain is NMDA receptor dependent. However, importantly, this major form LTP may be due to increased AMPA receptor density in the post-synaptic membrane and/or augmenting ion flow through the AMPA receptor [178,179].

EtOH consumption impairs learning and memory through the disturbance of NMDA receptor-dependent LTP and long-term depression (LTD), the plasticity signals related to memory processes in the hippocampus [155].

Pierrefiche and her colleagues previously showed in two EtOH binge-like episodes in young adult rats that (1) the LTD through the NMDA receptor is selectively blocked in hippocampal slices; (2) the NMDA receptor sensitivity to a GluN2B (NR2B) subunit antagonist is increased; and (3) cognitive deficits are induced [180]. Subsequently, they discovered that HSF2 is essentially involved in these three phenomena [181].

In the experiments for this discovery, they used WT- and *HSF2*-deficient mice (*HSF2*^−/−^ mice). Surprisingly, even under normal conditions (without EtOH), *HSF2*^−/−^ mice showed a selective loss of LTD in the hippocampus. This selective loss of LTD had a relationship with the phenomenon that the sensitivity of the NMDA-field excitatory postsynaptic potentials (fEPSPs) to the GluN2B antagonist was increased. During this experiment, the expression of the GluN2B subunit was increased in the *HSF2*^−/−^ mice compared to WT-mice; thus, the reason that the sensitivity of NMDA-fEPSPs to the GluN2B antagonist was increased may be due to the phenomenon that GluN2B expression was also increased in the *HSF2*^−/−^ mice [181]. These results suggest that HSF2 is required for proper glutamatergic synaptic transmission and LTD plasticity.

They performed the same experiment under the condition that mice can drink EtOH-containing water. In addition, they put two-bottles in a cage. One bottle contained only water. The other contained 10% EtOH in water (this experiment is called a two-bottle choice paradigm). After 1 month of chronic ethanol consumption, they performed mouse experiments. WT mice showed an increase in hippocampal synaptic transmission, an enhanced sensitivity to the GluN2B antagonist, and a blockade of LTD. In contrast, changes in synaptic transmission and plasticity were not found in *HSF2*^−/−^ mice.

From these results, they described that HSF2 is an important mediator of both glutamatergic neurotransmission and synaptic plasticity in basal conditions and also mediates ethanol-induced neuroadaptations of the hippocampus network after chronic ethanol intake.

**Significance of Section 7:**

HSF2 is a master HSF functioning during embryo growth.

HSF2-deficiency causes abnormalities in CNS development.

HSF2 is involved in FASD and its deficiency mitigates the influence of EtOH exposure.

HSF2 regulates glutamatergic synaptic transmission and LTD plasticity.

HSF2 is involved in synaptic plasticity disturbance caused by EtOH consumption.

## 8. Gene Bookmarking Is Indispensable Function of HSF2 in Cell Division, Cell Cycle, and Cell Proliferation

### 8.1. HSF2 Is the First Transcription Factor Demonstrated Its Gene Bookmarking Function

Restoring chromatin structure with high fidelity after cell division and the transcriptional reactivation of genes is critically indispensable for the survival of newborn daughter cells. During cell division, the genome is compacted in order for chromosomes to be segregated during cytokinesis. The condensin complex plays an important role in this compaction and is activated at the onset of mitosis.

However, a number of gene promoters, including *HSP70* gene promoter, are not tightly compacted in mitotic cells. The mechanism responsible for preventing compaction of specific gene regions during mitosis is called bookmarking [182]. Gene bookmarking factors interact with specific chromatin regions and this interaction ensure the restoration of the original gene expression pattern in daughter cells [183].

We recently summarized the importance of the discovery that HSF2 is the first transcription factor shown to have the gene bookmarking function [6]. This discovery must have accelerated the studies of the gene bookmarking mechanism, but the gene bookmarking system itself was recognized as a critical molecular mechanism indicated by publication of many review papers before Sarge’s group discovery [184,185].

Gene bookmarking has been observed in various gene promoters and transcription factors to date [183,186,187,188], and it is widely recognized that HSF2 is the first transcription factor demonstrated to bind to the gene promoter during mitosis and to be indispensable for gene bookmarking.

The gene bookmarking mechanism was proposed by Levens’ group in 1997 for the first time [189]. In mitosis, chromatin condenses, transcription is shut off, and most transcription factors are excluded from chromosomes [6,184,185]. However, before Levens’ group’s paper was published, several researchers discovered that chromatin is not completely condensed and that chromatin’s structure is disturbed; thus, some genes were revealed to be accessible for their transcription factors [190,191,192]. Levens and his colleagues showed that chromatin conformational distortion occurred on the TATA box region of the *HSP70*, *c-myc* and *beta-globin* promoters specifically during mitosis [189], but they could not show which transcription factors bind to these promoters in this paper.

### 8.2. HSP70 Gene Bookmarking of HSF2 Is Essential for Cell Survival Immediately after Cell Division

On the other hand, it was already shown that HSF2 can bind to the *HSP70* promoter, but no researchers could propose a persuasive explanation about the reason HSF2 binds to the *HSP70* promoter until the gene bookmarking role of HSF2 was discovered [193,194,195].

It was the year of 2005 that Sarge and his colleagues published a conclusive paper showing the gene bookmarking function of HSF2 [182]; they have been publishing the papers focused on the critical molecules for gene bookmarking since 1999 [196,197,198,199]. In the 1990s, it was gradually revealed that HSF has important functions for cell cycle progression [6,200,201]. Sarge’s group started a project to gain insight into the functions of HSF in cellular growth and cell cycle progression, discovered some important HSF2-interacting proteins, and finally reached the critically important discovery in 2005.

**Figure 2:**
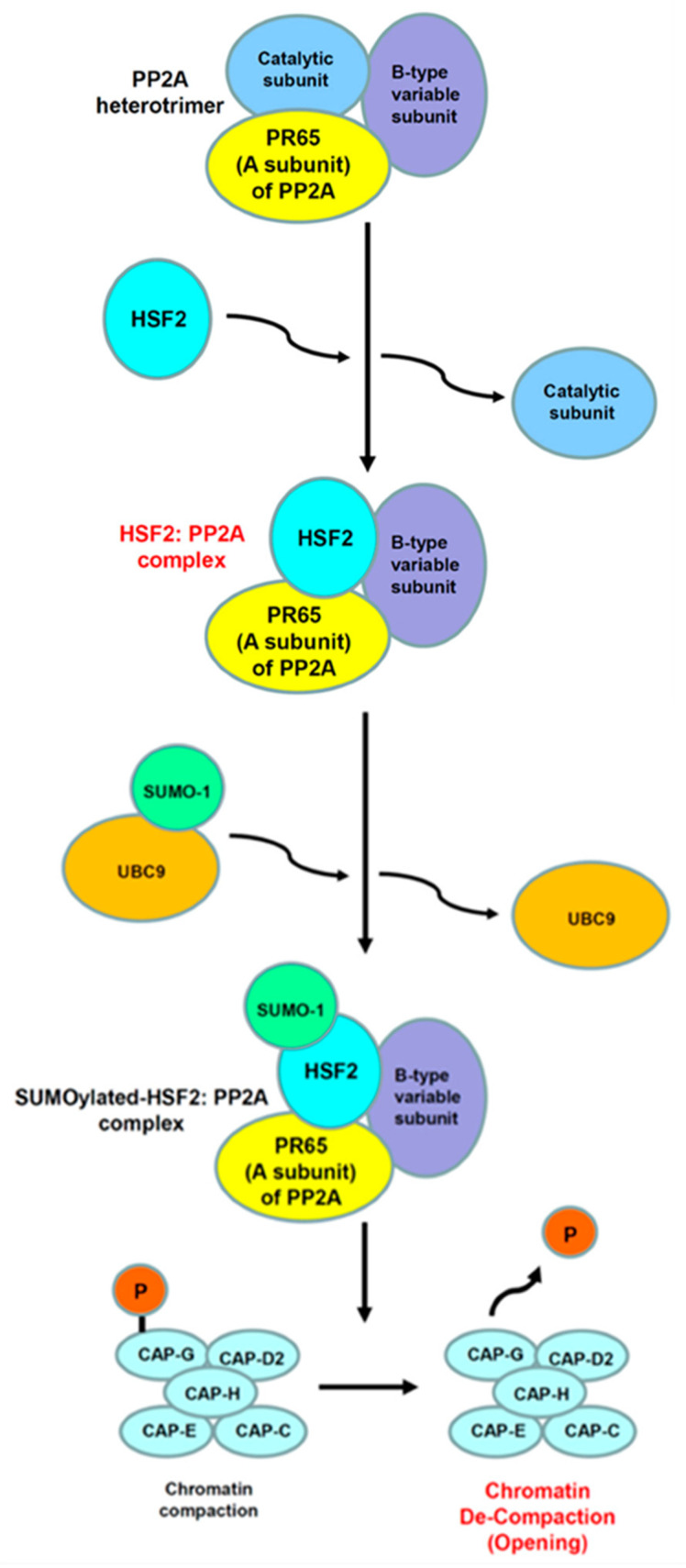
Simplified model of gene bookmarking by HSF2. This model is established on the HSP70 promoter [182]. However, this model probably comes into existence on other promoters.

As a first step, they performed a yeast two-hybrid screen using full-length mouse HSF1 and mouse HSF2. At this time, only HSF1 and HSF2 were identified and researchers did not have any clues as to which HSF is involved in cell division and cell cycle progression; thus, they probably tested both HSFs. Interacting proteins were screened by using human brain cDNA library. As a result, they found that HSF2 interacts with the PR65 (A subunit) of protein phosphatase 2A (PP2A) [196]. PP2A is involved in the regulation of a number of cellular processes including intermediary metabolism, signal transduction, and cell cycle progression by its dephosphorylating activity [202,203]. Additionally, PP2A is composed of a core heterodimer PR65 and catalytic subunit, and this core heterodimer interacts with a large number of B-type variable subunits. Finally, various types of PP2A heterotrimers exist in cells. When HSF2 interacts with PP2A, HSF2 competes with the catalytic unit, and the HSF2:PP2A complex is formed [196,197] (Figure 2).

In this yeast two-hybrid screen experiment, Sarge’s group discovered that another important protein interacts with HSF2. The protein is ubiquitin carrier protein 9 (UBC9), and UBC9 is well known as a SUMO-conjugating enzyme. This enzyme is encoded by the *UBE2I* gene; thus, another name is ubiquitin conjugating enzyme E2I (UBE2I). Different from ubiquitin, UBC9 does not have a role in protein degradation. Importantly, the fact that HSF2 interacts with UBC9 indicates that HSF2 is SUMOylated. Sarge and his colleagues performed several experiments, and demonstrated that HSF2 is SUMOylated and that Lys 82 residue is conjugated with SUMO-1 in vitro and in vivo [199]. SUMO-1 is a 97 amino acid and 11-kDa polypeptide (small protein), but the SUMO-1 modification has various functions. SUMO-1 modification at Lys 82 residue of HSF2 increased the DNA-binding activity of HSF2 [199].

Sarge and his colleagues discovered HSF2-interecting proteins other than UBC9 and SUMO-1 in this yeast two-hybrid screening. In these proteins, CAP-G was contained [182]. CAP-G is a subunit of condensin I (this condensin is also called as ‘canonical condensin complex’) [204,205]; as described, condensin complex plays an important role in genome compaction during mitosis and many genes become inactivated by this compaction.

They confirmed the interaction between HSF2 and CAP-G by immunoprecipitation. The interaction between HSF2 and SUMO-1 was similarly confirmed. In addition, they found that these interactions occur in only G_2_/M phase and not in G_0_/G_1_- and S- phases [182]. In other words, HSF2 associates with condensin in the G_2_/M phase and this association helps the binding to the *HSP70* promoter because HSP70 protein is indispensable for cell protection and survival in daughter cells. At the same time, SUMOylation of HSF2 contributes to making the binding of HSF2 to the *HSP70* promoter more reliable.

This group also found that HSF2 interacts with the PR65 (A subunit) of PP2A [196]. Condensin is consisted of five subunits, and CAP-G, CAP-D2, and CAP-H subunits requires phosphorylation for their activation [206]. PP2A has phosphatase activity; thus, PP2A can reverse the phosphorylation of CAP-G, CAP-D2, and CAP-H subunits. Sarge group showed that HSF2 associates with PP2A in mitotic cells and that PP2A exists on the *HSP70* promoter in mitotic cells [182]. It was also demonstrated that CAP-G, CAP-D2, and CAP-H subunits was dephosphorylated when the phosphorylated condensin was incubated with purified PP2A.

The data shown by Sarge and his colleagues in *Science,* published in 2005, suggested that HSF2 mediates *HSP70* promoter bookmarking by binding to this promoter in mitotic cells. In detail, HSF2 recruits PP2A and interacts with condensin through binding to CAP-G subunit. Recruited PP2A efficiently dephosphorylates condensin. Finally, condensin on this promoter (*HSP70* promoter) is inactivated, thereby compaction of this region of chromosomal DNA is prevented. This sequential molecular mechanism was shown in only one paper (Figure 2).

### 8.3. Now That the Gene Bookmarking Function Is Discovered in Various Transcription Factors

By the year 2022, the gene bookmarking mechanism was discovered in various genes, cells, and transcription factors. The gene bookmarking mechanism is now becoming recognized as a major molecular mechanism in cell division, daughter cell fate, and cell identity [188,207,208].

The gene bookmarking function always requires transcription factor, but for gene bookmarking, transcription factors also require forming complexes with other molecules. In the case of HSF2, it interacts with three condensin subunits, CAP-G, CAP-D2, CAP-H, TATA-binding protein (TBP), protein regulating cytokinesis 1 (PRC1), and PR65 (A subunit) of PP2A [182,196,206,209,210]. HSF2 interacts with PP2A in mitotic cells and that PP2A exists on the *HSP70* promoter in mitotic cells [63].

FoxA1 has also been shown to be a bookmarking transcription factor [188]. FoxA1 is involved in liver differentiation; in addition, it was reported that FoxA1 exhibited the greatest extent of mitotic chromosome binding [188].

Concerning liver differentiation, hepatocyte nuclear factor-4 alpha (HNF4alpha) and CCAAT/enhancer binding protein alpha (C/EBPalpha) have a gene bookmarking function. These transcription factors are required for the activation of most hepatic genes [211]. It has not been revealed what transcriptional complex FoxA1, HNF4alpha, and C/EBPalpha form when these three transcription factors act as gene bookmarking factors; however, we assume that they may interact with similar proteins to HSF2 and form similar complexes because the establishment of bookmarking must require interacting with condensin subunits in every transcription factor.

**Significance of Section 8:**

HSF2 is the first transcription factor demonstrated to have the function of gene bookmarking.

HSF2 gene bookmarks the *HSP70* gene during cell cycle progression.

HSF2 recruits PP2A and interacts with condensin through binding to the CAP-G subunit.

PP2A dephosphorylate condensin and this results in condensin inactivation. Consequently, compaction of this region of chromosomal DNA is prevented.

HSF2 forms transcriptional complexes for bookmarking.

It was discovered that some transcription factors including FoxA1, HNF4alpha, and C/EBPalpha have a bookmarking function.

## 9. Molecular Mechanisms of Transcriptional Activation of HSF2

### 9.1. HSF2 Interacts with HSF1 and Several HSPs

In general, transcription factors including HSFs are key regulators of biological processes and control the expression of their target genes by binding to transcriptionally regulatory regions, for example, promoters and enhancers [212]. In the case of HSF2, HSF2 binds to the specific heat shock element (HSE) sequence in various gene promoters, especially *HSP*s including *HSP70* and small HSP *alphaB-crystallin* (*CRYAB*) [2,57,62].

When transcription factors work, they always form their specific transcriptional complex. As already described, HSF2 forms complexes with condensin subunits and other proteins when functioning as a gene bookmarking factor [182].

HSF1 is known to form complexes with many various functional proteins so far. In 1995, Calderwood and his colleagues discovered that HSF1 forms a complex with heat shock cognate 70 protein (HSC70) in the cytoplasm of NIH-3T3 cells [213]. Consequently, Voellmy and his colleagues found that HSP90 interacts with HSF1 and discovered that this binding of HSP90 has a role to maintain HSF1 in inactivated state [139]. Their discoveries led to the demonstration of the molecular mechanism on how HSF1 is activated and inactivated.

HSF1 has now been revealed to form complexes with many proteins including HSP90, its cochaperone p23, FK506-binding protein 52 (FKBP52), p53, SirT1, replication protein A (RPA), ATF1, single-strand binding protein 1 (SSBP1), pericentromeric protein shugoshin 2, and estrogen receptor alpha (ERalpha) [214,215,216,217,218,219].

On the other hand, Yanagi and his colleagues discovered that the trimerization domain of human HSF2 interacts with nucleoporin p62 in 1997 [220]. Recently, Erkine and his colleagues performed interesting experiments. They replaced HSF with human HSF2 in yeast, and carried out high-throughput screening using this mutant yeast [221]. In this mutant yeast, human HSF2 was shown to interact with HSP70 and HSP90. In addition, overexpression of human HSF2 causes overexpression of HSP70 and HSP90, which is a likely cause of the slowed growth. In this first test, their new system discovered only two HSF2-binding and inhibiting molecules, HSP70 and HSP90. However, in the near future tests, they may discover more HSF2-binding molecules.

Unexpectedly, a few reports about HSF2-binding protein have been published. Under this circumstance, Mendillo and his colleagues tried to acquire more information about the relationship between HSF1 and HSF2 in cancer. They found that HSF2 physically and functionally interacts with HSF1 across diverse types of cancer [140]. In this paper, we already described (see Section 5.3), they finally suggested that HSF2 as a critical cofactor of HSF1 in driving a cancer cell transcriptional program to support the anabolic malignant state [140].

### 9.2. HSF2 and HSF1 Form Heterotrimer Complex and Activate Essential Genes in Specific Conditions

As already described, homotrimers of HSF2 and HSF1 interact in various gene promoters including several *HSP* genes [222]. HSF1 and HSF2 are co-expressed in numerous tissues and they can interact there; however, two groups independently discovered that HSF2 and HSF1 form heterotrimer [223,224]. Le Dréan and his colleagues showed that HSF2 and HSF1 form heterotrimers in the secreted protein chaperone *clusterin* gene [223]. Sistonen and her colleagues also showed that HSF2 and HSF1 form heterotrimers when bound to satellite III DNA in nuclear stress bodies, subnuclear structures in which HSF1 induces transcription [142,224]. The percentage of whole genes that are regulated by the HSF2-HSF1 heterotrimer are still unknown, though, we think that it is likely not strange that many genes are regulated by this heterotrimer because of structural homology between HSF2 and HSF1.

### 9.3. HSF2 Interacts with Set1/MLL Histone H3 Lysine 4 Methyltransferase Complex and Induces Gene Activation

As already indicated, a few reports about HSF2-binding protein unexpectedly have been published. In 2015, Hayashida and his colleagues showed that HSF2 binds to WDR5 in vitro and in vivo [2]. WDR5 has seven WD40 repeats, this repeat and WDR5 are well known as mediating protein–protein interactions [225,226].

**Figure 3:**
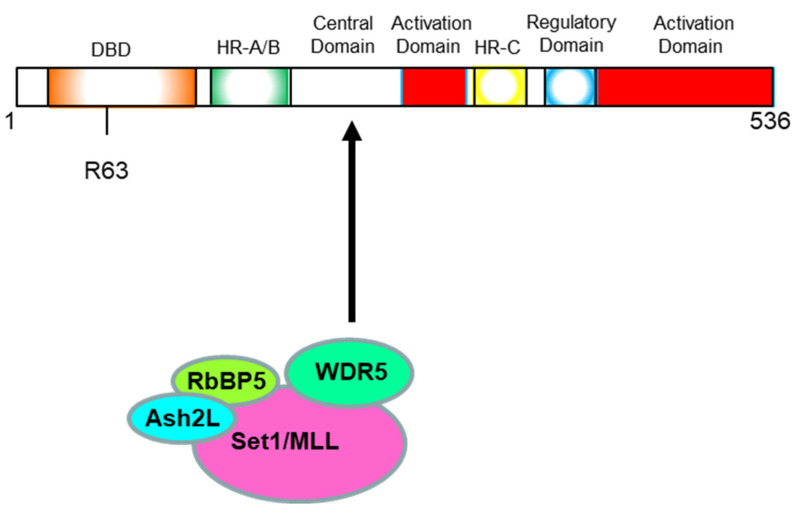
Interaction of HSF2 and Set1/MLL complex HSF2. Set1/MLL histone H3K4 methyltransferase and HSF2 probably form a large complex via WDR5 [2].

Hayashida reported that HSF2 directly binds to WD40 repeat protein WDR5, a core component of Set1 and Mixed Lineage Leukemia (MLL) H3K4 histone methyltransferase complex (Set1/MLL complex), and HSF2 and major components of Set1/MLL complex, WDR5, RbBP5, and Ash2L are recruited to the *CRYAB* promoter in the same behavior in MEF cells under normal and heat-stressed temperatures [2]. In HSF2-knockdown MEF cells, the relative amount of WDR5, RbBP5, Ash2L, Set1, MLL1, and MLL2 recruited to the *CRYAB* promoter were reduced under normal and/or heat-stressed conditions [2]. As the components of Set1/MLL complex have diverse functions, HSF2 may have various unrevealed functions with these components (Figure 3).

**Significance of Section 9:**

HSF2 directly binds to WD40 repeat protein WDR5.

Through the interaction with WDR5, HSF2 probably interacts with Set1/MLL histone H3.

Lys 4 methyltransferase complex (Set1/MLL complex).

HSF2 and HSF1 forms heterotrimer in specific genes including the secreted protein chaperone *clusterin* gene.

HSF2 and HSF1 also form heterotrimers when bound to satellite III DNA.

HSF2 may have various unrevealed functions with the components of Set1/MLL complex.

## 10. Conclusions and Perspectives

### 10.1. HSF2 Is Involved in Various Important Diseases through Its Functions

The purpose of writing this review paper was to summarize several findings about HSF2 and consider how much HSF2 is required for the survival of cells and living things through writing and summarizing.

Among four major mammalian HSFs, most research has been performed focused on HSF1. In this review paper, we indicated that HSF2 is a little involved in heat shock response, but HSF1, not HSF2, is a transcription factor pivotal in this response; however, we showed that HSF2 is deeply involved in various phenomena and many disorders in each section, as shown below:

– Polyglutamine Diseases (Section 3);
– Ulcerative Colitis (Section 4);
– Cancer (Section 5);
– Male Infertility (Section 6);
– Fetal Alcohol Spectrum Disorder (FASD) (Section 7).

It is not easy to reveal what roles HSF2 has in these different characterized diseases. Furthermore, HSF2 has a relationship with several kinds of cancers.

It is important to reveal the molecular mechanisms of how HSF2 causes these different diseases; however, this is not our purpose. Through the analysis of HSF2 or HSF2-interacting proteins, we must catch a clue in each disease at least within five years.

### 10.2. Is Gene Bookmarking the Most Important Function of HSF2?

We described the gene bookmarking mechanism and its importance in Section 8. The demonstration that HSF2 has a gene bookmarking function is a monumental work in cell division, cell cycle, and molecular biology without any doubt. Now that gene bookmarking transcription factors have been discovered, this gene bookmarking system is vital mechanism for us and other living things because this system makes it possible to reconstitute the same gene expression and epigenetics patterns. Without this system, different characteristics and different genes containing daughter cells are repeatedly born in every cell division, and finally, this species must be ruined.

We have many data about HSF2 which are not yet opened. We think that HSF2 will be involved in and required for the fundamental system of our survival, and its importance may be superior to HSF1. We expect that ongoing HSF2 research will bring us several more discoveries.

## Data Availability

Data sharing not applicable.
